# Deep Learning-Based Reconstruction of 3D Morphology of Geomaterial Particles from Single-View 2D Images

**DOI:** 10.3390/ma17205100

**Published:** 2024-10-18

**Authors:** Jiangpeng Zhao, Heping Xie, Cunbao Li, Yifei Liu

**Affiliations:** State Key Laboratory of Intelligent Construction and Healthy Operation and Maintenance of Deep Underground Engineering, College of Civil and Transportation Engineering, Shenzhen University, Shenzhen 518060, China; 13182389211@163.com (J.Z.); xiehp@szu.edu.cn (H.X.); cunbao.li@szu.edu.cn (C.L.)

**Keywords:** single-view 3D reconstruction, sand particle, deep learning, convolutional neural network

## Abstract

The morphology of particles formed in different environments contains critical information. Thus, the rapid and effective reconstruction of their three-dimensional (3D) morphology is crucial. This study reconstructs the 3D morphology from two-dimensional (2D) images of particles using artificial intelligence (AI). More than 100,000 particles were sampled from three sources: naturally formed particles (desert sand), manufactured particles (lunar soil simulant), and numerically generated digital particles. A deep learning approach based on a voxel representation of the morphology and multi-dimensional convolutional neural networks was proposed to rapidly upscale and reconstruct particle morphology. The trained model was tested using the three particle types and evaluated using different multi-scale morphological descriptors. The results demonstrated that the statistical properties of the morphological descriptors were consistent for the real 3D particles and those derived from the 2D images and the model. This finding confirms the model’s validity and generalizability in upscaling and reconstructing diverse particle samples. This study provides a method for generating 3D numerical representations of geological particles, facilitating in-depth analysis of properties, such as mechanical behavior and transport characteristics, from 2D images.

## 1. Introduction

**Figure 1:**
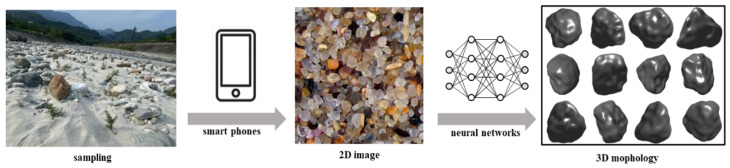
Schematic of the 3D reconstruction of natural sand particles using a single-view 2D image.

Geomaterials, such as soil and rock, consist of particles whose morphology significantly influences the material’s physical, mechanical, and thermal properties [1,2]. Three-dimensional (3D) particle representations are required for physical and mechanical analyses of geomaterials [3,4]. However, obtaining 3D representations of microscopic geomaterial particles is challenging. Effective methods, such as computed tomography (CT) [5,6], white light interferometry [7], and 3D laser scanning [8], are employed in laboratories to conduct high-resolution 3D analyses of the particles’ morphology. However, these methods are costly, labor intensive, and time-consuming, making them inconvenient for routine use, especially when in situ 3D information is required. In contrast, high-resolution two-dimensional (2D) images can be easily obtained from other microscopy techniques or a smartphone at a fraction of the cost (less than 1% of the 3D imaging costs). It is well-known that 2D information is a projection of the 3D structure [9], encoding the 3D data within a lower-dimensional form. A skilled observer can intuitively reconstruct the 3D morphology of an object from a 2D image. Therefore, reconstructing 3D morphology from 2D images is a solvable problem. Figure 1 illustrates the concept of reconstructing the 3D morphology of natural sand particles from a single-view 2D image.

The reconstruction of particles’ 3D morphologies yields numerical entities representing fundamental elements in discrete element modeling (DEM) [10] and digitized particles. Several attempts have been made to reconstruct 3D morphology using 2D information. Wang [11] reconstructed the 3D morphology of rock particles using multi-view 2D images. However, acquiring multiple views in real-world scenarios is challenging, and the generalization performance may not be sufficient. Some researchers have conducted detailed analyses on reconstructing the 3D pore structure of rock bodies using 2D slices [12,13]. Reconstructing 3D pores is fundamentally different from reconstructing particles’ 3D morphologies. Genetic algorithms (GAs) [14] have been used in DEM simulations to reconstruct particles’ morphologies [15,16], but this method is only applicable to clumps of spheres and requires some 3D information [17]. Some researchers have employed generative models like variational autoencoders (VAEs) [18] for 3D morphology reconstruction [17,19]. However, these approaches generate additional 3D particles without addressing the dimensional upscaling from two to three dimensions.

Due to the rapid development of artificial intelligence (AI) in recent years, deep learning methods [20] have improved significantly. They can learn implicit high-dimensional mapping relationships from large amounts of data [21,22], making them well-suited for 2D to 3D mapping tasks. Multi-view approaches facilitate the reconstruction of 3D morphology [23,24]; the fewer the views, the more challenging the reconstruction, with single-view reconstruction being the most difficult [25,26]. Predicting the complete 3D shape of an object from a single image is a long-standing and extremely challenging task. Recently, several representations for 3D models, including point cloud [27], mesh [28], and signed distance field [29], have been adopted in 3D objects. A deep learning architecture, the pixels-to-voxels model (Pix2Vox) proposed by Xie [30,31], can reconstruct 3D object morphology from single views. However, it cannot reconstruct the complex 3D morphology of natural particles. For single-view reconstruction of particles, current challenges include a lack of representative real datasets, research on the upscaling and 3D reconstruction of natural particles is limited, and efficient algorithms and models with high reconstruction speed and quality for reconstructing the morphology of natural particles are lacking, and the absence of work for reconstructing particles from a single view.

Therefore, deep learning has significant potential for reconstructing the 3D morphology of particles from 2D single-view images. The 3D representation of particles primarily includes voxel, point cloud, and mesh formats, each requiring different data preprocessing methods and models for training. Given the regularity of voxel representation, which integrates well with Convolutional Neural Networks (CNNs) and is widely used in deep learning [32,33], our study employs voxels to express 3D particles. We propose the pixels-to-voxels of particles (PVP) model based on the Pix2Vox for reconstructing the 3D morphology of irregular sand particles from 2D images. We construct a dataset comprising more than 100,000 particles. The results indicate that the 3D morphology reconstructed from 2D images closely matches the real 3D morphology, demonstrating the model’s suitability for dimensional upscaling.

## 2. Materials and Methods

### 2.1. Dataset

Data are critical in deep learning approaches. The quantity and quality of the data significantly affect model performance [34]. A sufficiently large and low-noise dataset enables the model to learn the intrinsic data patterns. Generally, data augmentation and data balancing techniques are used when there is a lack of sufficient and balanced data [35,36].

**Figure 2:**
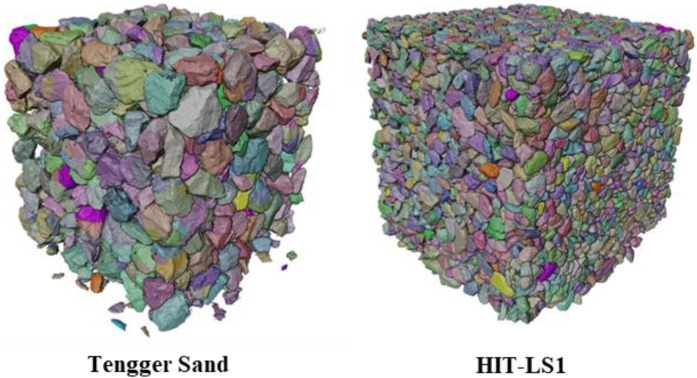
The µCT scans of the samples.

This study utilized three types of samples: naturally formed particles (Tengger sand), manufactured particles (HIT-LS1 lunar soil simulant) [37], and numerically generated digital particles. We determine the 3D morphology of the samples by scanning them sequentially using micro CT (µCT), achieving ultra-high resolution with voxel dimensions of 2048 × 2048 × 2048 and a data volume exceeding 40 GB. Figure 2 shows the µCT scans of the samples. The final dataset included 25,000 particles of the HIT-LS1 lunar soil simulant (Dataset 1), 58,000 particles of the Tengger sand (Dataset 2), and 25,000 numerically generated digital particles (Dataset 3). The total dataset comprised more than 100,000 particles, with 90% of the particles used as the training set and 10% as the test set.

### 2.2. Network Architecture

**Figure 3:**
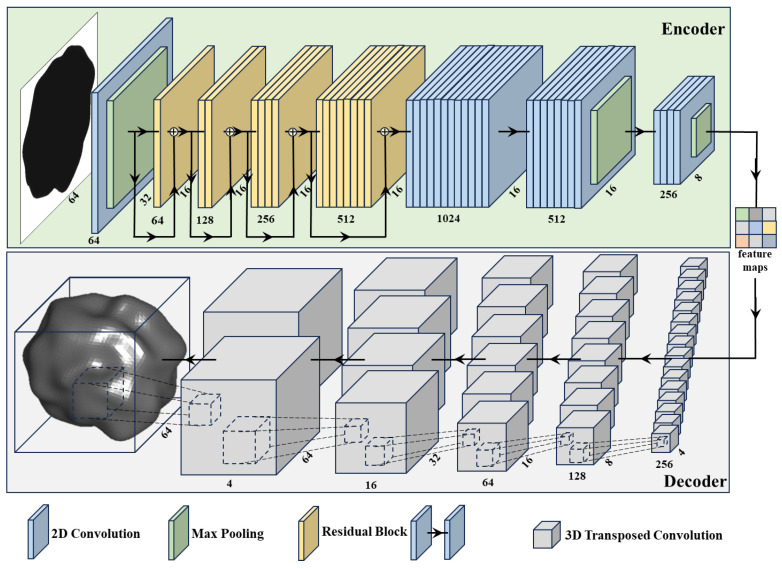
The network architecture of the PVP model.

The PVP model was designed to reconstruct the 3D morphology of particles from a single 2D grayscale image. The model’s architecture is based on the classic deep learning model Pix2Vox++ [28], but was modified to handle irregular, randomly oriented particles. The 3D morphology was randomly projected to generate the input 2D grayscale images, and no fixed viewpoints were used, enhancing the model’s ability to learn mapping features. The architecture of the PVP (Figure 3) consists of an encoder and a decoder. The encoder extracts features from the input 2D grayscale image for encoding. The decoder generates the corresponding 3D voxels from the extracted features. Due to memory constraints and the need for effective visualization of the particles, the voxel resolution was 64^3^. The 2D image size was 64^2^ pixels to ensure consistency between input and output.

The calculation formulas for the encoder and decoder of the autoencoder are briefly outlined in [38], and here we provide a detailed formula for the encoder, which includes 2D convolutional layers, max pooling layers, and residual connections:

The equation for the 2D convolution is as follows:

(1) $$y[i,j]=\sum_{m=0}^{k-1}\sum_{n=0}^{k-1}x[i+m,j+n]\cdot w[m,n]+b$$

The equation for the max pooling is as follows:

(2) $$y[i,j]=\underset{m,n}{\max}\{x[i+m,j+n]\}$$

The equation for the residual connection is as follows:

(3) $$y=F(x,\{W_{i}\})+x$$

where x[i+m,j+n] represents the input feature map pixel values, w[m,n] represents the convolutional kernel weights, b represents the bias, y[i,j] represents the output feature map pixel values. $W_{i}$ represents the weights, and $F(x,\{W_{i}\})$ represents the output after applying convolution and other operations to the input x.

The equation for the 3D transposed convolution in the decoder is as follows:

(4) $$y[e,f,g]=\sum_{m=0}^{k-1}\sum_{n=0}^{k-1}\sum_{l=0}^{k-1}x[e+m,f+n,g+l]\cdot w[m,n,l]+b$$

where x[e+m,f+n,g+l] represents the input feature map voxel values, w[m,n,l] represents the convolutional kernel weights, b represents the bias, and y[e,f,g] represents the output feature map voxel values.

#### 2.2.1. Encoder

The encoder extracts features from the input image, which are used to reconstruct the 3D morphology of the particles. The input image passes through multiple layers: a convolutional layer with a 3^2^ convolution kernel (followed by batch normalization and ReLU activation), a 2^2^ pooling layer, four residual blocks (each containing two convolutional layers and a residual connection), two convolutional layers with a 3^2^ convolution kernel, a 2^2^ pooling layer, a convolutional layer, and a 2^2^ pooling layer. The feature dimensions are upscaled from 64 to 1024 to capture specific features, followed by downscaling to 256, producing a 4^2^ feature map. This architecture is inspired by the classic Visual Geometry Group (VGG) algorithm [39], and is tailored to the sizes of the particle output features. The inclusion of residual blocks [40] helps to prevent vanishing features and allows for deeper networks, enhancing the model’s expressive power.

#### 2.2.2. Decoder

The decoder converts the feature map extracted by the encoder into voxels. The decoder comprises six 3D transposed convolution layers with a 4^3^ convolution kernel (followed by batch normalization and ReLU activation) to map the features into voxels. Dropout layers are added between the middle two 3D transposed convolution layers to prevent overfitting and improve generalization. The final layer does not have a batch normalization layer and uses a Sigmoid activation function to produce voxel values, downscaling the feature dimensions from 512 to 1 and resulting in the reconstructed 64^3^ voxel representation.

In the encoder, the process of handling a single input image involves extracting features and increasing dimensionality from an image sized 64^2^ × 1 through 2D convolution, ultimately transforming it into 4^2^ × 256 feature maps, totaling 256. These feature maps are then input into the decoder, where they are first converted to a voxel size of 2^3^ × 512 with a dimension of 512. Subsequently, 3D transposed convolution is employed to reconstruct the final output as a 64^3^ × 1 3D model.

### 2.3. Loss Function

Different architectures and data types require appropriately tailored loss functions to help the model learn the data features. We used the Adam optimization algorithm to minimize the loss function and maximize the similarity between the reconstructed and real particles. The loss function is defined as follows:

(5) $$Loss=10\cdot {Loss}_{BCE}+{\lambda \cdot Loss}_{MSE}$$

The final loss consists of two parts $Loss_{BCE}$ represents the binary cross-entropy loss, a common and effective loss function for binary classification. It is used to classify the predicted voxel values as 0 or 1. λ is a weighting parameter that adjusts the contribution of ${Loss}_{MSE}$. The $Loss_{BCE}$ is defined as follows:

(6) $$Loss_{BCE}=\frac{1}{N}\sum_{i=1}^{N}\left[r_{i}\log(p_{i})+(1-r_{i})\log(1-p_{i})\right]$$

where ${Loss}_{MSE}$ represents the mean squared error loss, which is commonly used to evaluate the difference between the predicted and actual values. It is a standard approach in regression problems. The ${Loss}_{MSE}$ is defined as follows:

(7) $$Loss_{MSE}=\frac{1}{N}\sum_{i=1}^{N}{\left(r_{i}-p_{i}\right)}^{2}$$

where N represents the number of voxels in the real particle, $r_{i}$ represents the voxel value for the real particle, and $p_{i}$ represents the voxel value of the predicted particle at the same position. $Loss_{BCE}$ and ${Loss}_{MSE}$ decrease as the predicted particle approximates the real particle. A smaller loss indicates better reconstruction results.

### 2.4. Evaluation Indicators

**Figure 4:**
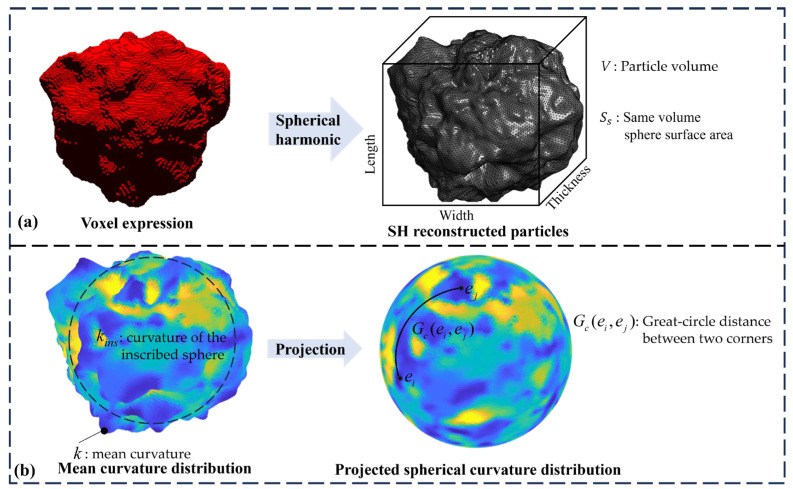
(**a**) The voxel is represented using spherical harmonic reconstruction into Fibonacci triangular grid particles. (**b**) The mean curvature distribution and projected curvature distribution of particles.

It is impossible to reconstruct the complex and disordered 3D morphology of microscopic particles using a single 2D image with limited information. Instead, we evaluated the reconstruction effectiveness by selecting parameters characterizing particles of the same type. Voxel representation facilitates model learning and training. We used spherical harmonic (SH) reconstruction of the voxel particles to eliminate the impact of voxel roughness on parameter calculation [41], as illustrated in Figure 4a.

Extensive research has been conducted on particle characterization [9,42] to evaluate the reconstruction performance. We calculated the triaxial dimensions (length, width, thickness) and basic morphological parameters, including the elongation index, volume, and surface area [6,43,44] based on SH reconstruction. Additionally, we incorporated effective and complex characterization parameters, such as sphericity (S), roundness (R), and structural index (SI), to quantify particle morphology. These were calculated as follows:

(8) $$S=\frac{S_{s}}{S_{A}}=\frac{\sqrt[{3}]{36\pi V^{2}}}{S_{A}}$$

(9) $$R=\frac{\sum k^{-1}}{N_{e}k_{ins}^{-1}}$$

(10) $$E_{pb}=\frac{1}{2}\sum_{i=1}^{N_{c}}\sum_{j=1,j\neq i}^{N_{c}}\frac{1}{G_{c}(e_{i},e_{j})}$$

(11) $$SI=\frac{E_{pb}}{E_{p}}$$

where V represents the particle volume, $S_{s}$ denotes the surface area of a sphere with the same volume, and $S_{A}$ is the particle’s surface area. k is the mean curvature of the original particle surface, $k_{ins}$ is the curvature of the inscribed sphere, $N_{e}$ is the number of points where k exceeds $k_{ins}$, and R is obtained by weighted averaging. The roundness ranges from 0 to 1; the closer it is to 1, the more spherical and smoother the particle, with fewer sharp corners (local maximum curvature). $e_{i}$ and $e_{j}$ represent positions on the sphere, and $G_{c}(e_{i},e_{j})$ refers to the great circle distance between two corners, with $N_{c}$ representing the number of corners. $E_{p}$ is the Riesz energy, an indicator of uniformity, and $E_{pb}$ is the Riesz energy for the spherical Fibonacci distribution of $N_{c}$ points, representing the minimum energy [19]. A detailed description can be found in Figure 4b.

## 3. Results and Comparison with Other Models

### 3.1. Ablation Study

This section analyzes the contributions of various components within the model through an ablation study, aiming to assess the impact of hyperparameters and model architecture on performance. The hyperparameters are crucial in deep learning. They help the model to learn effectively and enhance its generalization capability. Hyperparameters refer to parameters that must be defined before training, such as the coefficient of the loss function (if applicable), learning rate, batch size, network architecture, and training steps. We focused on the most critical hyperparameters, i.e., the loss function coefficient and learning rate, because they have the greatest impact on the model’s performance. All other hyperparameters had standard values, as shown in Table 1 (Num_workers is number of threads used by the model during data loading and processing). The hyperparameter analysis was conducted using 25,000 particles from the HIT-LS1 lunar soil simulant (Dataset 1).

#### 3.1.1. Loss Function

**Figure 5:**
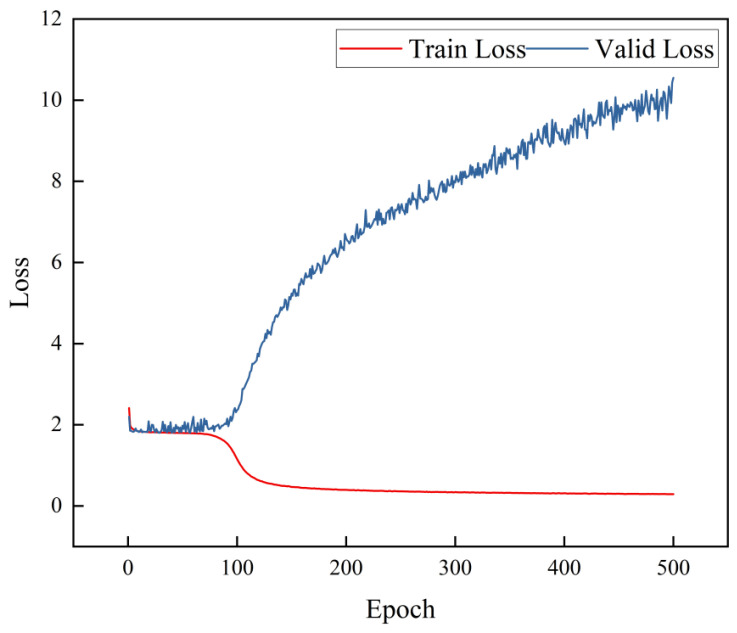
Loss function curve for the Classic Pix2Vox++ model applied to the HIT-LS1 particles.

We conducted experiments using the ${Loss}_{p2v}$ loss function of the classic Pix2Vox++ model. The loss curve is shown in Figure 5.

(12) $${Loss}_{p2v}=10\cdot {Loss}_{BCE}$$

The training and validation losses do not decrease significantly during the first 100 steps. The training loss decreases after 100 steps, but the validation loss increases rapidly, exceeding the initial loss. This result indicates that the model only fits the training data. The validation loss diverges significantly, suggesting overfitting. Therefore, the classic Pix2Vox++ model is not suitable for reconstructing particles.

**Figure 6:**
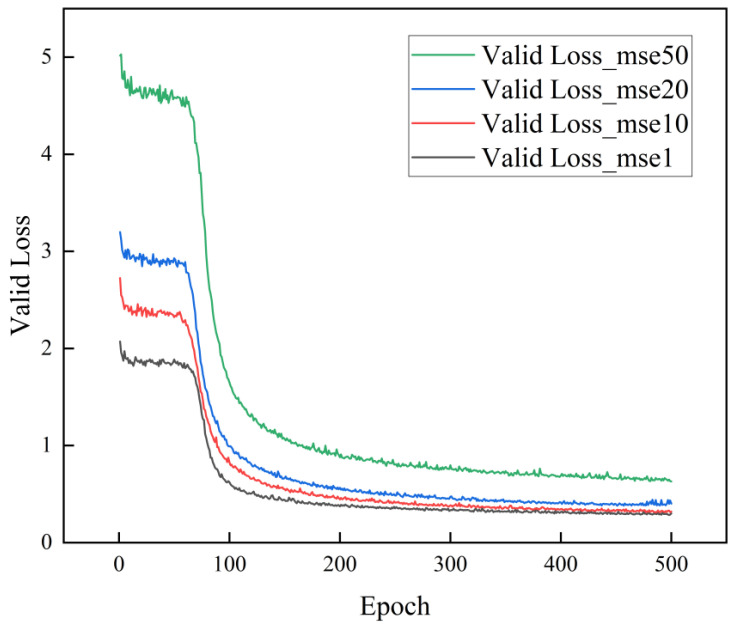
Loss curves of models with different λ values.

We improved the loss function using Equation (1) and experimented with different values of λ (50, 20, 10, 1) to determine the coefficient of $Loss_{BCE}$. The trends in the validation loss for different numbers of iterations and λ values are shown in Figure 6. The model converges when different λ values are used. As λ decreases, the convergence result decreases. However, as the value approaches 10, it is very close to the result at 1. Therefore, the optimum convergence effect is achieved when λ is 1.

#### 3.1.2. Learning Rate

The learning rate has the most significant impact on the model’s loss. A low learning rate increases the convergence time, and a high rate may lead to incomplete convergence, causing the model to fall into a local minimum or gradient explosion. We tested learning rates of 0.05, 0.005, and 0.0005 and implemented a variable learning rate strategy. We began at 0.005 and reduced the learning rate by 95% every ten epochs until it reached 0.0004.

**Figure 7:**
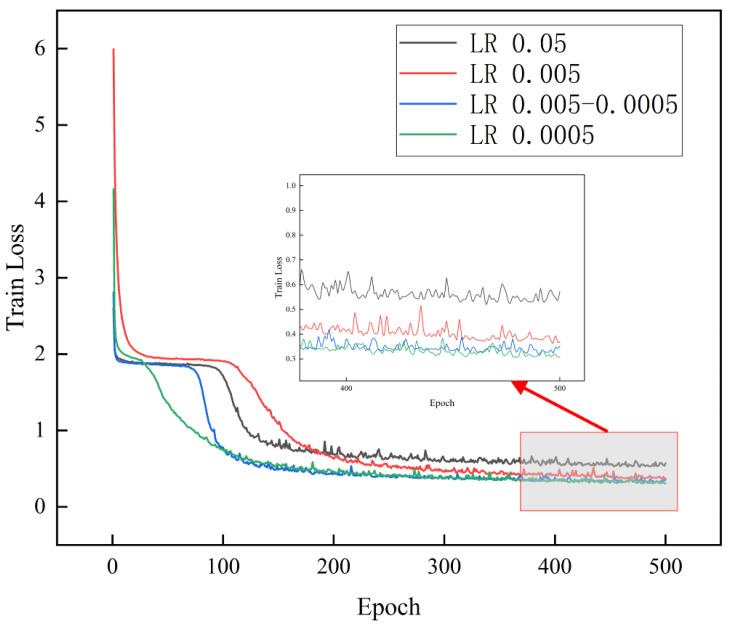
Model loss curves with different learning rates (LR).

As shown in Figure 7, the loss at convergence is the smallest, with a learning rate of 0.05. As the learning rate decreases, the loss at convergence decreases, but the difference between 0.005 and 0.0005 is minimal. Therefore, the optimal convergence is around 0.0005. Although the convergence at 0.0005 is the smallest, it converges more slowly. The blue line representing the variable learning rate ensures that the initial convergence avoids local minima while achieving the best final convergence result. Thus, we selected a variable learning rate, starting at 0.005, decreasing by 95% every ten epochs.

### 3.2. Reconstruction Performance of Different Models

#### 3.2.1. Lightweight Model

The proposed PVP model did not exhibit underfitting; however, it is unclear whether overfitting occurs and whether the model complexity matches the data volume. Therefore, we reduced the size of the PVP model by half to reduce its complexity. The small PVP (PVP-S) model was used to analyze whether it matched the data. The PVP model’s encoder was reduced from 15 to 7 layers, and the decoder was reduced from 6 to 4 layers. The residual blocks and 2D convolutional layers for feature extraction were retained to obtain the feature map, and the main structure of the 3D transposed convolutions and dropout regularization was the same. This reduction reduced model size from 385 M to 134 M.

#### 3.2.2. Comparison of Results from Different Models

**Figure 8:**
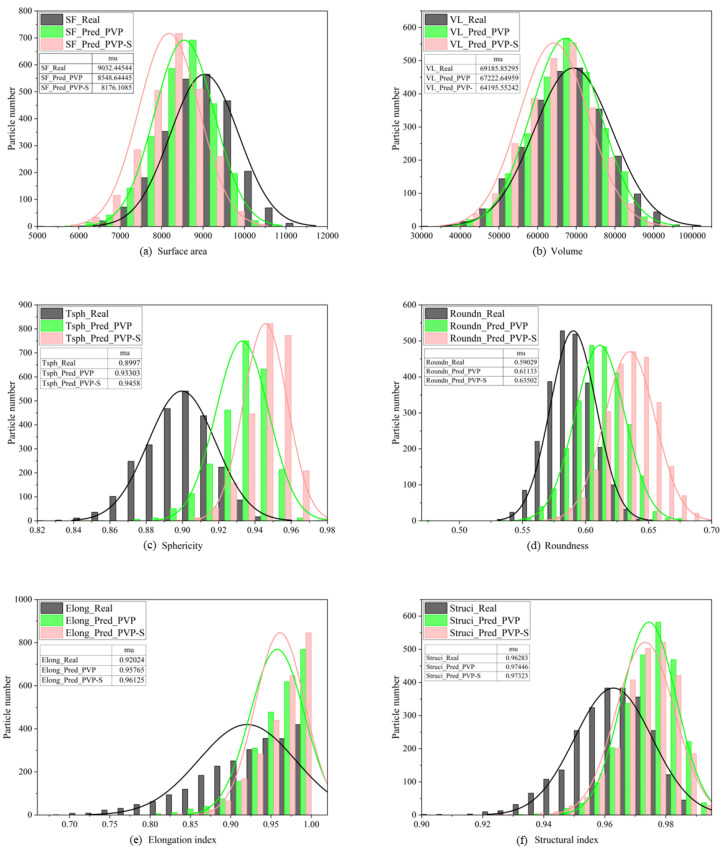
Comparison of evaluation indicators of the reconstruction performance of two models for the HIT-LS1 dataset.

The reconstruction results of the PVP and PVP-S models for the training set were evaluated using 2495 particles. Figure 8 presents the histogram of the real and reconstructed particles obtained from different models. Real: real particles, Pred PVP: predicted particles using the PVP model, and Pred PVP-S: predicted particles using the PVP-S model.

As shown in Figure 8a,b, similar distributions of the surface area and volume are obtained from both models, indicating that they accurately reconstruct the morphological features of the particles. The differences between the average values of the evaluation indicators for the real and reconstructed particles for the PVP and PPVP-S models are listed in Table 2. The difference in the surface area is significantly smaller for the PVP model than for the PVP-S model. The difference is more pronounced for the volume. The distribution of the sphericity (Figure 8c) and roundness (Figure 8d) is similar for the reconstructed and real particles, and the PVP model performs better than the PVP-S model. The elongation index (Figure 8e) and structural index Figure 8f) exhibit less satisfactory results, but are acceptable. Neither model shows an advantage based on these metrics. However, the small differences in their average values suggest that the distributions of the two statistical parameters are similar.

The results demonstrate that the PVP model outperforms the PVP-S model, showing a good match of the model to the dataset. This finding indicates that the PVP model learns the morphological characteristics of the particles and can reconstruct the 3D morphology from a single 2D image. Although it cannot capture fine texture details, it successfully learns the features, resulting in a similar distribution of the metrics for the reconstructed and real particles.

### 3.3. Reconstruction Results for Natural Sand Particles and Numerically Generated Digital Sand Particles

We used the PVP model to compare the evaluation indicators for the real Tengger sand (Dataset 2), and the numerically generated digital particles (Dataset 3) to assess the model’s generalization ability and suitability in reconstructing 3D morphology from a single-view 2D image. The results demonstrates the model’s performance for analyzing different particle samples with significant differences. A total of 2462 particles were used for testing.

**Figure 9:**
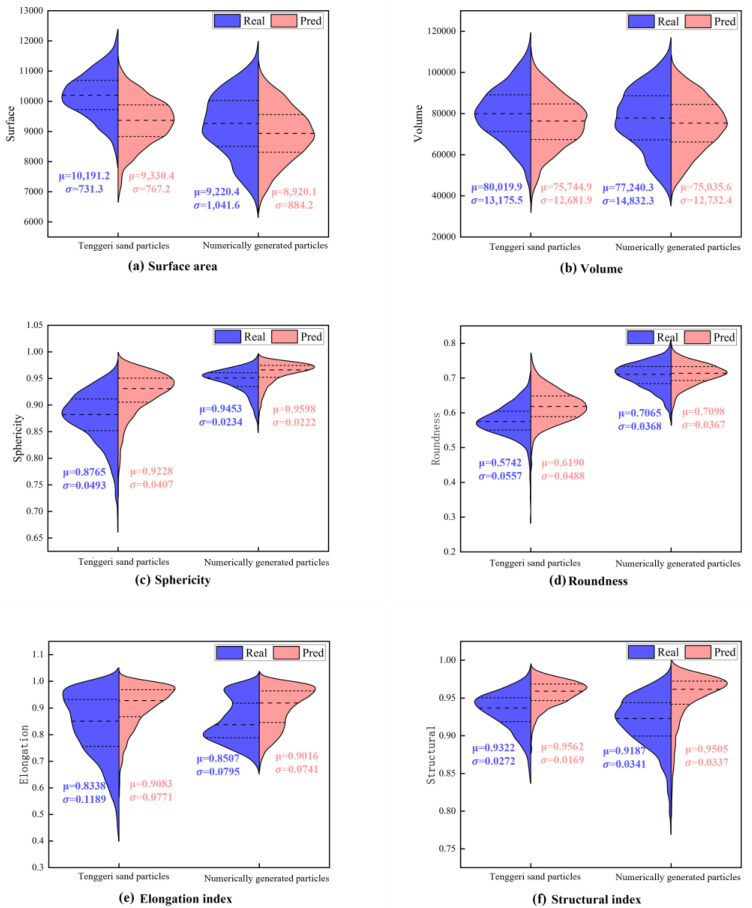
Six violin plots of evaluation indicators for natural and numerically generated sand particles.

Figure 9 shows the violin plots of the evaluation indicators for the real and model-reconstructed particles. The violin plots represent the probability distribution of the parameters, with the vertical axis showing the parameter values and the horizontal width representing the number of particles at each value. The left violin plot depicts the distribution of the Tengger sand particles, and the right one shows the distribution of the numerically generated particles. Blue represents the real particles, and red represents the model-reconstructed ones.

Figure 9a,b shows the data for the surface area and volume, respectively. The distribution is similar for the reconstructed and real groups. As shown in Figure 9a, the surface area is smaller for the reconstructed Tengger sand, indicating a worse performance than for the numerically generated particles. The reconstruction results for the volume are similar for both samples (Figure 9b), demonstrating excellent results. The sphericity (Figure 9c) and roundness (Figure 9d) show significant differences. The values are smaller for Tengger sand, and the distribution is broader than for the numerically generated sand. The distributions of both reconstructed samples closely resemble that of the real particles, although the sphericity has slightly larger values. The roundness values are slightly less accurate for Tengger sand than for the numerically generated particles. The elongation index for the real Tengger sand and the numerically generated sand exhibits a bimodal distribution, which is not perfectly aligned with the reconstructed group (Figure 9e). Figure 9e,f shows that the distribution of the real group is broader, and that of the reconstructed group is more concentrated.

**Figure 10:**
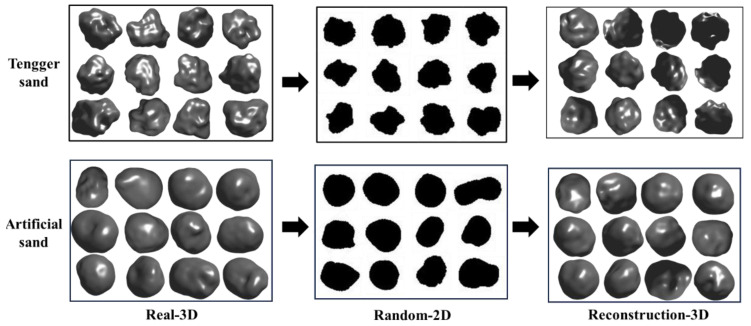
Three-dimensional morphology of the real Tengger sand and the numerically generated sand particles, 2D random projections, and PVP-reconstructed 3D morphology.

Figure 10 presents the 3D morphology of the real particles, their 2D random projections, and the PVP-reconstructed 3D morphology for the Tengger sand and numerically generated particles. The Tengger sand exhibits a more complex morphology, whereas the numerically generated digital particles are smoother. The reconstruction results reflect these differences, indicating that the model can adapt to particle samples with significant differences in the distribution. Although the model demonstrates good generalization ability, the reconstruction of the real samples is less realistic than that of the numerically generated digital samples.

## 4. Discussion

We compared multiple evaluation indicators obtained from different models and particle sample types. The morphological parameters of the real particles exhibited a more dispersed distribution. Since deep learning models learn from data, all features are derived from the dataset. The numerically generated digital particles are highly characterized and parameterized, resulting in distinctive morphology and the best learning results. The morphological features of natural sand are formed by random complex processes like hydrodynamics, weathering, and fragmentation. Thus, these features are more challenging to learn due to high complexity and noise. The lunar soil simulant was processed (crushing, grinding, sintering, etc.) and has more uniform features, resulting in better learning outcomes than natural sand but worse results than for the numerically generated digital particles.

We used the PVP model for 2D single-view reconstructions of 3D particle morphologies. Training with a sample size of less than 50,000 particles required only 10 h on an NVIDIA 4090 GPU. The trained model reconstructed 2500 particles in approximately 4 s. Unlike traditional parameter-based methods that are limited to statistical representation and analysis of parameters without producing numerical entities, our model can upscale and reconstruct the morphology of irregular particles. The reconstructed particle distributions were generally consistent with the real distributions, and the model quickly generated large quantities of 3D numerical particles. It can be used to create particle libraries for input into DEM simulations.

This approach is particularly suitable for reconstructing particles that cannot be analyzed using 3D CT experiments, such as real lunar soil particles, which are rare, but whose 2D images are abundant. With the continuous advancement of deep space exploration, humanity will access more celestial bodies, allowing for the acquisition of richer geomaterials’ 2D images. This enables the reconstruction of 3D morphologies from single 2D images of asteroids or rocks, significantly aiding space exploration and showcasing substantial potential.

Moreover, our model has some inherent limitations. While the voxel data representation is structurally simple and facilitates the learning of overall features, it contains excessive internal useless data, causing the model to learn not only surface features, but also waste computational resources on useless internal data. Furthermore, memory constraints necessitate the use of lower resolutions. Other data representations, such as point clouds and meshes, hold more advantages; however, there are currently no related models for single-view reconstruction of particles, and the training difficulty is greater, making the validity of results uncertain. Therefore, this paper does not include experimental comparisons with other deep learning methods.

## 5. Conclusions

The objective of this study was to develop a rapid AI model for reconstructing 3D morphology from a single-view 2D image of particles. We used a dataset of more than 100,000 particles of HIT-LS1 lunar soil simulant, Tengger sand, and numerically generated digital particles. Transfer learning techniques were used, leveraging the backbone network of the Pix2Vox model, and different models were trained. The following conclusions were obtained:

1. The distributions were similar for the reconstructed and real particles for the three sample types, indicating that upscaling from a single-view 2D image to 3D morphology was statistically feasible.
2. The PVP model provided distributions of the reconstructed particles consistent with the real distributions. The surface area and volume were highly similar. The similarity between the distributions of the reconstructed and real particles for natural and numerically generated particles demonstrated the strong generalization ability of the model and its suitability for different particle types.
3. Due to differences in formation, the reconstruction results were better for the HIT-LS1 lunar soil simulant than for the natural sand, but worse for the numerically generated sand particles, reflecting varying levels of difficulty for the AI model.

Reconstructing 3D particle morphology from a single-view 2D image overcomes the limitations of parameter-based particle characterization. It provides numerical elements for the assessment of 3D morphology and a robust and effective sample database for DEM. However, due to the memory limits of the voxel data format, alternative formats, like point clouds and frequency domain data, could be utilized to overcome it and achieve closer alignment with the distribution of real particles.

## Figures and Tables

**Table 1:** Some model hyperparameters.

Epoch	Learning Rate	Batch Size	Optimizer	Num_Workers
500	0.005	256	Adam	12

**Table 2:** The differences between the average evaluation indicator values for the real and reconstructed particles for the PVP and PVP-S models.

	Surface Area	Volume	Sphericity	Roundness	Elongation Index	Structural Index
PVP	**5.3%**	**2.8%**	**3.7%**	**3.6%**	**4.0%**	1.3%
PVP-S	9.5%	7.2%	5.1%	7.6%	4.5%	**1.1%**

## Data Availability

The original contributions presented in the study are included in the article, further inquiries can be directed to the corresponding author.
